# Inhale, exhale: Why particulate matter exposure in animal models are so acute? Data and facts behind the history

**DOI:** 10.1016/j.dib.2019.104237

**Published:** 2019-07-08

**Authors:** Flávio Curbani, Fernanda de Oliveira Busato, Maynara Marcarini do Nascimento, David Nicholas Olivieri, Carlos Eduardo Tadokoro

**Affiliations:** a Programa de Pós-Graduação em Ecologia de Ecossistemas, Universidade Vila Velha, Rua Comissário José Dantas de Melo, 21, Boa Vista, CEP 29102-920, Vila Velha, ES, Brazil; b Departamento de Tecnologia Industrial, Centro Tecnológico, Universidade Federal do Espírito Santo, Av. Fernando Ferrari, 514, Goiabeiras, CEP 29060-970, Vitória, ES, Brazil; c Laboratory of Immunobiology, Universidade Vila Velha, Rua Comissário José Dantas de Melo, 21, Boa Vista, CEP 29102-920, Vila Velha, ES, Brazil; d Programa de Pós-Graduação em Ciências Farmacêuticas, Universidade Vila Velha, Rua Comissário José Dantas de Melo, 21, Boa Vista, CEP 29102-920, Vila Velha, ES, Brazil; e Escuela Superior de Ingeniería Informatica, University of Vigo, Spain

**Keywords:** Concentration, Dose, Health effects, Inhalation rate, Instillation, Toxicology

## Abstract

We present a dataset obtained by extracting information from an extensive literature search of toxicological experiments using mice and rat animal models to study the effects of exposure to airborne particulate matter (PM). Our dataset covers results reported from 75 research articles considering paper published in 2017 and seminal papers from previous years. The compiled data and normalization were processed with an equation based on a PM dosimetry model. This equation allows the comparison of different toxicological experiments using instillation and inhalation as PM exposure protocols with respect to inhalation rates, concentrations and PM exposure doses of the toxicological experiments performed by different protocols using instillation and inhalation PM as exposure methods. This data complements the discussions and interpretations presented in the research article “Inhale, exhale: why particulate matter exposure in animal models are so acute?” Curbani et al., 2019.

**Table:** Specifications table

Subject area	*Environmental Science*
More specific subject area	*Air quality, particulate matter, toxicology and health*
Type of data	*Table*
How data was acquired	*Data acquired by literature search and normalization of data from different exposure protocols based on a model that calculates concentration, inhalation rates and particulate matter exposure dose. The selection filters applied to build the dataset were: published works and data were published in 2017, in the English language, and were referenced in indexed journals (with editorial board, peer reviewed and included in Clarivate Analytics Journal Citation Reports). Also, the papers selected were based on mice and/or rat models, and the protocol exposed the respiratory tract to PM to study the health effects in one or more specific endpoints (respiratory tract, pulmonary and extra-pulmonary). Given these conditions, a search string was used to query PubMed Particularly, the following keywords were used: (particulate matter) AND (mice or mouse or rats or rat) AND (inhalation or instillation). Apart from the most recent articles published in 2017, we also included seminal papers from previous years.*
Data format	*Calculated and analysed*
Experimental factors	*Data were compiled from experimental design description from 75 different research articles (155 toxicological experiments)*
Experimental features	*We used a model to calculate inhalation rates, concentrations and PM exposure dose from 155 different toxicological experiments*
Data source location	*Vila Velha, Brazil, Universidade Vila Velha*
Data accessibility	*Data is provided with this article*
Related research article	F. Curbani, F.O. Busato, M.M. Nascimento, D.N. Olivieri, C.E. Tadokoro, Inhale, exhale: why particulate matter exposure in animal models are so acute? Environmental Pollution. In Press [1]

**Table:** 

**Value of the data** • The data is valuable to researchers interested in understanding the health effects of particulate matter by means of experimental studies with animal models. • The variables described are the concentration, dose, and inhalation rate used in different toxicological experiments with mice and rats performed by instillation and inhalation. • This data consists of a normalized collection of different experimental protocols of exposure to particulate matter that is useful for comparing experiments findings. • Normalization of the exposure variables allows the ability to compare results to reported environmental particulate matter concentrations. • Comparisons between the results from different exposure protocols and comparison with environmental concentrations allows for a better understanding of the exposure protocols that can improve experimental design of future studies.

## 1 Data

The data consists of a list of selected articles where particulate matter (PM) exposure experiments were performed in mice and rats. From each of source article, we extracted the objectives of each study as related by the original authors (Table 1). The compiled data and normalization were processed with an equation based on a PM dosimetry model considering physiological breathing parameters and PM inhaled fraction (inhalability) in mice and rats (Table 2). From the collection of selected articles, the data provides a list of reviewed experiments, methods and characteristics of each exposure protocol, including concentrations, doses and PM inhalation rates normalized by a PM dose model (Table 3), and a summary of the variables from different PM exposure protocols in mice and rats (Table 4).

**Table 1:** List of articles from PubMed where PM exposure was performed in mice and rats and the objectives of each study as related by the authors. The dataset includes 30 articles published in 2017 and 45 articles published in previous years.

Exposure protocols	Objectives	Authors
CAP inhalation	The objective of this study was to determine whether short-term exposures to concentrated ambient particles alter the morphology of small pulmonary arteries in normal rats and rats with chronic bronchitis (CB).	[15]
CAP inhalation	Our objective was to provide experimental plausibility for epidemiological observations by testing the hypothesis that exposure to particulate matter with nominal mean aerodynamic diameters of 2.5 μm or less (PM_2.5_) during discrete periods of pregnancy results in preterm birth (PTB) and low birth weight (LBW).	[16]
CAP inhalation	The objectives of the present study were: 1) to determine whether acute exposure to low levels of particles promotes measurable acute systemic and cardiopulmonary effects; and 2) to assess if the magnitude of the observed alterations is influenced by season.	[17]
CAP inhalation	This study investigates the effects of inhaled ammonium sulphate, which is a major compound of inorganic air pollutants in PM_2.5_, on adult neurogenesis in aged Sprague-Dawley rats.	[18]
CAP inhalation	The hypothesis tested was that older animals would exhibit more severe pulmonary inflammation and haematological changes following the CAP exposure when compared to young, normal animals.	[19]
CAP inhalation	We evaluated the effects of air pollution on the adrenal cortex using female mice. One group was conditioned daily in a chamber with exposure to particulate matter.	[20]
CAP inhalation	Mice were used to investigate the effects of iRhom2 on PM_2.5_-induced hepatic dyslipidaemia.	[21]
CAP inhalation	The present work was designed to: (i) determine whether short-term exposure to concentrated air particles causes pulmonary inflammation in normal rats; (ii) characterize the component(s) of CAP that are significantly associated with the development of the inflammatory reaction; and (iii) define the induction of mediators and other pathophysiological response elements of the lung with CAP exposure.	[22]
CAP inhalation	The aim of this study was to investigate the mechanism by which PM_2.5_ influences the Notch signalling pathway leading to worsening immune disorder and accelerating chronic obstructive pulmonary disease (COPD) development.	[23]
CAP inhalation	We investigated the roles of T-helper (Th)1–Th2 cytokines and nasal remodelling after ambient PM_2.5_ exposure in a rat model of allergic rhinitis.	[13]
CAP inhalation	We investigated the in vivo effects of PM_2.5_ exposure on the inflammatory response, oxidative stress, the enzyme activities of Na + K + -ATPase and Ca2+-ATPase, and the morphology and function of mitochondria in the nasal mucosa of rats.	[24]
CAP inhalation	To evaluate the ability of particulate air pollution to promote oxidative stress and tissue damage in vivo, we studied a rat model of short-term exposure to concentrated ambient particles.	[25]
CAP inhalation	We conducted a study to compare the inflammatory response of the lung to instilled versus inhaled particles.	[26]
CAP inhalation	We have investigated whether long-term inhalation exposure to diesel engine exhaust, a dominant contributor to particulate air pollution in urban environments, can aggravate Alzheimer's Disease (AD)-like effects in female 5X Familial AD (5XFAD) mice and their wild-type female littermates.	[27]
CAP inhalation	The aim of this study was to identify the impact of titanium dioxide (TiO_2_) nanoparticles on inflammasome in a mouse model of allergic asthma.	[28]
CAP inhalation	To test the impact of chronic airborne particulate matter exposure on the upper respiratory system in vivo.	[12]
CAP inhalation	To determine whether oxidants are implicated in PM-dependent lung inflammation, we tested the ability of N-acetylcysteine (NAC) to prevent lung inflammation in a rat model of short-term exposure to concentrated ambient particles.	[29]
CAP inhalation	The objectives of this study were (1) to determine whether short-term exposures to concentrated air particles cause pulmonary inflammation in normal rats and rats with chronic bronchitis (CB); (2) to identify the site within the lung parenchyma where CAP-induced inflammation occurs; and (3) to characterize the component(s) of CAP that is significantly associated with the development of the inflammatory reaction.	[30]
CAP inhalation	The objective was to identify and quantify estrogenic receptor-b (ERb), aryl hydrocarbon receptor (AhR), the cytochrome P450 enzymes CYP1A1, 1A2, 1B1, and mucus profile in the nasal epithelium of mice	[31]
CAP inhalation	This study evaluated the inflammatory differences in BALB/c mouse males and females in three phases of the estrous cycle that were exposed to ambient air or concentrated ambient particles.	[32]
CAP inhalation	The purpose of this study was to determine the respiratory effects of inhaled ultrafine iron particles in rats.	[33]
CAP inhalation and intranasal instillation	We compared the physiological consequences of short-term exposure to diesel exhaust via inhalation to those due to exposure to the same diesel exhaust particles suspended in solution and delivered intranasally.	[34]
CAP inhalation and intratracheal instillation	The present study was designed to compare intratracheal instillation to inhalation exposure derived health endpoints of acute lung toxicity in the rat that relate to homologous clinical outcomes that have been reported with ambient PM using a well characterized model emission PM, which would have demonstrable and relevant effects at low lung doses by both methods.	[35]
CAP inhalation and intratracheal instillation	We used pharmacological strategies to determine whether oxidants are implicated in PM-dependent cardiac dysfunction and whether PM-induced increase in autonomic stimulation on the heart mediates cardiac oxidative stress and toxicity.	[36]
EAP inhalation	The aim of this study was to verify the effects of ambient air pollution of São Paulo City on coronary of healthy non-isogenic Swiss mice, chronically exposed since birth until adulthood.	[37]
EAP inhalation	We investigated effects of chronic exposure (2 months) to ambient levels of particulate matter on development of protease-induced emphysema and pulmonary remodelling in mice.	[38]
EAP inhalation	The aim of the present study was to analyse the effects of air pollution in the city of São Paulo on mouse female fertility.	[39]
EAP inhalation	The present study was conducted to a) determine whether short-term exposure to ambient levels of particulate air pollution from vehicles elicits inflammatory responses and lipid peroxidation in rat lungs, and b) determine if intermittent short-term exposures induce some degree of tolerance.	[40]
Intranasal instillation	To investigate how the combination of soybean allergens and diesel exhaust particles (DEP) can affect the induction or exacerbation of asthma in a murine model.	[41]
Intranasal instillation	We hypothesized that sub-chronic exposure to PM_2.5_ in HFD-treated mice, susceptible to type 2 diabetes mellitus (T2DM), would also be able to change nutrient metabolism-related tissues (NMRT) cellular antioxidant defense, and the balance between intracellular 70-kDa heat shock proteins (iHSP70) and extracellular 72-kDa heat shock proteins (eHSP72) contents expressed as [eHSP72]/[iHSP70] ratio, predisposing for a major risk of cellular damage and development T2DM.	[42]
Intranasal instillation	We assessed the effects of Bufei Huoxue (BFHX) capsules on PM_2.5_-induced pulmonary inflammation and the underlying mechanisms of action.	[43]
Intranasal instillation	This study aimed to investigate the effects of winter and spring particulate matter on airway inflammation and allergies in a mouse asthma model.	[44]
Intranasal instillation	This study aimed to investigate the effects of AD on the early stage of antigen sensitization using a mouse model of asthma, as well as the role of leukotrienes (LTs) in antigen-induced airway inflammation potentiated by AD particles.	[45]
Intranasal instillation	In order to assess the relationship between PM_2.5_ exposure and autism spectrum disorder, neonatal male Sprague–Dawley rats were chosen and exposed to PM_2.5_ by intranasal instillation.	[46]
Intranasal instillation	The aim of this work was to evaluate the time changes of systemic markers of oxidative stress and inflammation, after an acute exposure to Residual Oil Fly Ash (ROFA).	[47]
Intranasal instillation	Our objective was to analyse air PM from downtown Buenos Aires (UAP-BA) and evaluate its biological impact on normal airways. We studied the inflammatory response to intranasal instillation of UAP-BA in a short-term-exposure mouse model.	[48]
Intranasal instillation	We studied lung responses to low doses of urban air particulate matter from Buenos Aires (UAP-BA), with special emphasis on oxidative balance.	[49]
Intranasal instillation	The objective was to verify how these organic compartments respond to increasing concentrations of particles of known elemental composition.	[50]
Intranasal instillation	The aim of this study was to analyse in vivo the acute biological impact of two environmental particles, urban air particles from Buenos Aires and Residual Oil Fly Ash, on the cardiorespiratory system of middle-aged mice, evaluating oxidative metabolism and inflammation.	[51]
Intranasal instillation	We tested the hypothesis that a single acute exposure to low doses of fine particulate matter (PM_2.5_) may induce functional and histological lung changes and unchain inflammatory and oxidative stress processes. PM_2.5_ was collected from the urban area of São Paulo city during 24 h and underwent analysis for elements and polycyclic aromatic hydrocarbon contents.	[52]
Intranasal instillation	The aim of this study was to evaluate the effects of subchronic exposure to low doses of diesel exhaust particles (DEP) instilled in the respiratory tract of mice.	[53]
Intranasal instillation	The therapeutic effects of stemonine on mice with PM_2.5_-induced COPD were investigated in the present study.	[54]
Intranasal instillation	To test our hypothesis that cardiovascular diseases associated with sulphur dioxide (SO_2_), nitrogen dioxide (NO_2_), or PM_2.5_ exposure are the result of increased heart rate (HR), decreased blood pressure (BP) and enhanced systemic inflammation.	[55]
Intratracheal instillation	The aim of the present study was to evaluate the effects of PM_10_ on electrocardiogram (ECG) parameters, blood pressure, lipid peroxidation (MDA), xanthine oxidase, and antioxidant enzyme in healthy rats and also to examine the protective effects of vanillic acid (VA) in this respect.	[56]
Intratracheal instillation	This study aims to observe whether the combined treatment with vitamin E (vit E) and omega-3 polyunsaturated fatty acids (U-3 FA) could prevent the fine particulate matter (PM_2.5_)-induced cardiovascular injury through alleviating inflammation and oxidative stress.	[57]
Intratracheal instillation	The purpose of our study is to investigate PM_10_ sum effects on lungs and extra pulmonary tissues. The aim of this study is to disclose the pulmonary short-term effects and extra-pulmonary translocation of PM_10_ sum collected in Milano urban centre.	[58]
Intratracheal instillation	To assess susceptibility to lung infection following coexposure to particulate matter.	[59]
Intratracheal instillation	In this study, we evaluated the primary oxidative stress produced in the lung by crystalline silica (SiO_2_) in the early phase after SiO_2_ exposure. The aim of this study is to understand the crystalline SiO_2_-induced pulmonary oxidative stress in the early phase.	[60]
Intratracheal instillation	This study was conducted to investigate the possible protective effects and mechanisms of aspirin, Vitamin C, Vitamin E, or ozone on fertility in female mice treated with PM_2.5_.	[61]
Intratracheal instillation	We investigated the association of the chemical composition and sources of urban air fine (PM2.5−0.2) and coarse (PM10−2.5) particulate samples with the inflammatory activity in the mouse lung.	[62]
Intratracheal instillation	This study was undertaken to clarify the effects of Asian sand dust on lung eosinophilia in mice immunized beforehand by ovalbumin (OVA).	[63]
Intratracheal instillation	In the present study, urban PM2.5 and coarse particulate matter (CPM) collected during haze events of Northeast China in the winter season were used. The exacerbating effects of PM_2.5_ and CPM on OVA-induced allergic inflammation in murine lungs were compared to clarify the role of the chemicals and microbial materials in the two types of PM.	[64]
Intratracheal instillation	In order to further understand the roles of microRNAs in regulating the imbalance of T-helper 1 (Th1)/T-helper 2 (Th2) differentiation triggered by PM_2.5_.	[65]
Intratracheal instillation	The current study aimed to evaluate the effects of size-fractioned PM on lung immune responses in healthy BALB/c mice.	[66]
Intratracheal instillation	we investigated whether exposure to PM_2.5_, a PM with an aerodynamic diameter of less than 2.5 mm, enhances inflammation-related toxicity in the human respiratory system through activation of the epidermal growth factor receptor (EGFR) signalling pathway.	[67]
Intratracheal instillation	This study investigated the effect of acute respiratory exposure to PM on eyes, as induction of retinal thickening.	[68]
Intratracheal instillation	We investigated whether PM instillation in the airway could alter the course of acute lung injury, using a murine model with experimental lung injury induced by intratracheal lipopolysaccharide (LPS) challenge.	[69]
Intratracheal instillation	The objective is to investigate the influence of PM_2.5_ exposure on peripheral blood lymphocyte subsets in pregnant mice and the antagonism of quercetin on adverse effects induced by PM_2.5_ exposure.	[70]
Intratracheal instillation	We intend to investigate the toxic effects of PM_2.5_ during summer and winter on reproductive cells and tissues and focus on endoplasmic reticulum stress (ERS) to illustrate the possible molecular mechanisms.	[71]
Intratracheal instillation	We wished to investigate the impact of PM_2.5_ on placenta and prenatal outcomes and its related mechanisms in a rat model.	[72]
Intratracheal instillation	We assessed the effect of prolonged exposure to diesel exhaust particles (DEP) on chronic renal failure induced by adenine, which is known to involve inflammation and oxidative stress.	[73]
Intratracheal instillation	To evaluate the effect of airborne particulate matter 2.5 (PM_2.5_) in winter on airway inflammation, water-soluble supernatant (Sup) and water-insoluble precipitate (Pre) in PM_2.5_ were inoculated in NC/Nga mice with high sensitivity to mite allergens.	[74]
Intratracheal instillation	To evaluate the allergic effect of airborne particulate matter (PM) on the airway, separated soluble supernatant (Sup) and insoluble precipitate (Pre) in suspended PM were inoculated into NC/Nga mice with a high sensitivity for mite allergens.	[75]
Intratracheal instillation	The allergic inflammatory effects of particulate matter PM_2.5_, collected with the cyclone system in Yokohama city in Japan, were investigated in NC/Nga mice.	[2]
Intratracheal instillation	We aimed to explore the toxic mechanisms of cardiovascular injuries induced by ambient fine particulate matter (PM_2.5_) in atherosclerotic-susceptible ApoE−/− mice.	[76]
Intratracheal instillation	We investigated by the optical microscopy some cytological characteristics of the bronchoalveolar lavage fluid cell population 24 h after intratracheal instillation of microscale manganese dioxide (MnO_2_) and barium chromate (BaCrO_4_) particles (separately or together at two different doses) into the lungs of Wistar rats.	[77]
Intratracheal instillation	The aim of this study is to disclose short-term adverse effects on respiratory and cardiovascular systems induced by winter fine particles exposure.	[78]
Intratracheal instillation	The immune cells, including pulmonary macrophages of Sprague–Dawley (SD) rats and Raw 264.7 cells, were applied to further investigate the effect of PM_2.5_ on cell autophagy of macrophages, thus clarified the possible molecular mechanism of immunotoxicity caused by PM_2.5_.	[79]
Intratracheal instillation	We hypothesized that mechanisms independent of inflammation contribute to accelerated thrombus formation following exposure to diesel exhaust particles (DEP).	[80]
Intratracheal instillation	The primary objective of this study was to provide insights on the factors affecting the toxicological potency of exhaust PM emitted from different light-duty vehicles. This study presents different research techniques linked together to improve our understanding of the particulate matter (PM) impacts on health. The study develops conceptual dose–response functions for the different vehicle configurations.	[81]
Intratracheal instillation	In order to understand the comprehensive pulmonary response to PM_2.5_ stress, a non-targeted high-throughput metabolomics strategy was adopted to characterize the overall metabolic changes and relevant toxicological pathways.	[82]
Intratracheal instillation	We constructed a rat model to investigate the roles of autophagy in blood-testis barrier (BTB) toxicity induced by PM_2.5_. Sprague–Dawley rats were developmentally exposed to normal saline (NS) or PM_2.5_ with the doses via intratracheal instillation.	[83]
Intratracheal instillation	Short- and long-term exposure to particulate matter (PM) 2.5 instigates adverse health effect upon the cardiovascular system. We demonstrated that Wuhan PM_2.5_ exposure induced elevation of systemic Angiotensin II (ANGII) and local angiotensin-converting enzyme (ACE)/ANGII/ANGII type 1 receptor (AT1R) axis activation and the subsequent oxidative stress and proinflammatory responses in the vascular endothelium.	[84]
Intratracheal instillation	In order to investigate the mechanisms in PM_2.5_ toxicity, we explored the endogenous metabolic changes and possible influenced metabolic pathways in rats after intratracheal instillation of PM_2.5_.	[85]
Intratracheal instillation	The aim of this study was to evaluate the inflammatory response to SiO_2_ nanoparticles using in vivo test systems.	[86]

EAP: environmental air PM, CAP: concentrated air PM.

**Table 2:** Physiological breathing parameters and PM inhaled fraction (inhalability) in mice and rats.

Variables	Mouse	Rat	Authors
Body mass, *Bw* (kg)	0.025	0.250	[6]
Tidal volume, *V*_*t*_ (mL)	0.218	2.100	[7]
Minute volume, *V*_*m*_ (mL/min)	60.4	252.0	[7]
Breathing frequency, *f* (min^−1^)	277	120	[7]
Inhalability, *I* (%)			
PM_1_	88	92	[4]
PM_2.5_	67	75	[4]
PM_10_	20	25	[4]

**Table 3:** List of experiments where PM exposure was performed in mice and rats, with methods and characteristics of each procedure. The dataset includes 30 articles published in 2017 and 45 articles published in previous years.

Exposure method	PM size	Animal model	Equivalent atmospheric concentration (μg/m^3^)	Time of one exposure event (h)	Number of exposure events	Total exposure time (h)	Inhaled dose per event (μg/kg *Bw*)	PM inhalation rate (μg/kg *Bw*/h)	Authors
EAP inhalation	PM_2.5_	Mouse	18.1	2880	1	2880	12619.2	4.4	[37]
	PM_10_	Rat	22.0	6	1	6	5.0	0.8	[40]
	PM_10_	Mouse	33.9	24	60	1440	58.9	2.5	[38]
	PM_10_	Rat	34.0	6	1	6	7.7	1.3	[40]
	PM_10_	Mouse	48.9	24	120	2880	85.0	3.5	[39]
	PM_10_	Rat	99.2	5	4	20	18.7	3.7	[40]
	PM_10_	Rat	112.4	20	1	20	85.0	4.2	[40]
	PM_10_	Rat	138.6	20	1	20	104.8	5.2	[40]
	PM_10_	Rat	224.7	6	1	6	51.0	8.5	[40]
CAP inhalation	PM_1_	Mouse	50.0	2	3	6	31.9	15.9	[28]
	PM_1_	Rat	57.0	6	3	18	47.6	7.9	[33]
	PM_1_	Rat	90.0	6	3	18	75.1	12.5	[33]
	PM_2.5_	Mouse	60.9	6	80	480	88.7	14.8	[12]
	PM_2.5_	Mouse	101.5	6	120	720	147.8	24.6	[21]
	PM_2.5_	Mouse	113.4	6	17	102	165.2	27.5	[16]
	PM_2.5_	Rat	126.1	5	3	15	71.5	14.3	[15]
	PM_2.5_	Rat	126.1	5	3	15	71.5	14.3	[30]
	PM_2.5_	Mouse	163.8	6	17	102	238.6	39.8	[16]
	PM_2.5_	Rat	170.7	5	3	15	96.8	19.4	[15]
	PM_2.5_	Rat	170.7	5	3	15	96.8	19.4	[30]
	PM_2.5_	Rat	187.1	5	3	15	106.1	21.2	[15]
	PM_2.5_	Rat	187.1	5	3	15	106.1	21.2	[30]
	PM_2.5_	Rat	200.0	3	30	90	68.0	22.7	[24]
	PM_2.5_	Rat	200.0	3	30	90	68.0	22.7	[13]
	PM_2.5_	Mouse	203.0	1	6	6	49.3	49.3	[17]
	PM_2.5_	Mouse	203.3	1	6	6	49.3	49.3	[17]
	PM_2.5_	Rat	262.2	5	3	15	148.7	29.7	[22]
	PM_2.5_	Rat	267.3	5	3	15	151.6	30.3	[15]
	PM_2.5_	Rat	267.3	5	3	15	151.6	30.3	[30]
	PM_2.5_	Rat	300.0	1	1	1	34.0	34.0	[25]
	PM_2.5_	Rat	300.0	3	1	3	102.1	34.0	[25]
	PM_2.5_	Rat	300.0	5	1	5	170.1	34.0	[25]
	PM_2.5_	Rat	300.7	5	3	15	170.5	34.1	[15]
	PM_2.5_	Rat	300.7	5	3	15	170.5	34.1	[30]
	PM_2.5_	Rat	400.0	6	3	18	272.2	45.4	[19]
	PM_2.5_	Rat	481.0	5	3	15	272.7	54.5	[15]
	PM_2.5_	Rat	481.0	5	3	15	272.7	54.5	[30]
	PM_2.5_	Rat	595.0	2	28	56	134.9	67.5	[18]
	PM_2.5_	Mouse	600.0	1	12	12	145.7	145.7	[32]
	PM_2.5_	Mouse	600.0	1	21	21	145.7	145.7	[20]
	PM_2.5_	Mouse	600.0	1	12	12	145.7	145.7	[31]
	PM_2.5_	Rat	700.0	5	1	5	396.9	79.4	[36]
	PM_2.5_	Mouse	770.0	1	90	90	186.9	186.9	[23]
	PM_2.5_	Mouse	950.0	6	15	90	1383.7	230.6	[27]
	PM_2.5_	Mouse	950.0	6	65	390	1383.7	230.6	[27]
	PM_2.5_	Rat	1000.0	3	30	90	340.2	113.4	[24]
	PM_2.5_	Rat	1000.0	3	30	90	340.2	113.4	[13]
	PM_2.5_	Rat	1228.0	5	10	50	696.3	139.3	[29]
	PM_2.5_	Rat	2000.0	3	30	90	680.4	226.8	[13]
	PM_2.5_	Rat	3000.0	3	30	90	1020.6	340.2	[24]
	PM_2.5_	Rat	12000.0	6	1	6	8164.8	1360.8	[35]
	PM_2.5_	Mouse	20000.0	2	8	16	9710.1	4855.0	[34]
	PM_2.5_	Mouse	30000.0	2	8	16	14565.1	7282.6	[34]
	PM_10_	Rat	100.0	6	20	120	22.7	3.8	[26]
	PM_10_	Rat	1000.0	6	20	120	226.8	37.8	[26]
	PM_10_	Rat	10000.0	6	20	120	2268.0	378.0	[26]
Intranasal instillation	PM_2.5_	Mouse	700.3	1	9	9	170.0	170.0	[49]
	PM_2.5_	Mouse	700.3	1	9	9	170.0	170.0	[48]
	PM_2.5_	Mouse	823.9	1	1	1	200.0	200.0	[52]
	PM_2.5_	Mouse	823.9	1	84	84	200.0	200.0	[42]
	PM_2.5_	Mouse	2471.7	1	1	1	600.0	600.0	[52]
	PM_2.5_	Mouse	4119.4	1	1	1	1000.0	1000.0	[51]
	PM_2.5_	Mouse	4119.4	1	1	1	1000.0	1000.0	[47]
	PM_2.5_	Mouse	4119.4	1	7	7	1000.0	1000.0	[55]
	PM_2.5_	Mouse	4943.3	1	21	21	1200.0	1200.0	[53]
	PM_2.5_	Mouse	4943.3	1	42	42	1200.0	1200.0	[53]
	PM_2.5_	Mouse	13841.3	1	8	8	3360.0	3360.0	[34]
	PM_2.5_	Mouse	20762.0	1	8	8	5040.0	5040.0	[34]
	PM_2.5_	Rat	17636.7	1	14	14	2000.0	2000.0	[46]
	PM_2.5_	Mouse	24716.6	1	9	9	6000.0	6000.0	[41]
	PM_2.5_	Mouse	41194.4	1	28	28	10000.0	10000.0	[55]
	PM_2.5_	Mouse	164777.4	1	4	4	40000.0	40000.0	[43]
	PM_2.5_	Mouse	164777.4	1	7	7	40000.0	40000.0	[54]
	PM_2.5_	Rat	176366.8	1	14	14	20000.0	20000.0	[46]
	PM_10_	Mouse	55.2	1	1	1	4.0	4.0	[50]
	PM_10_	Mouse	552.0	1	1	1	40.0	40.0	[50]
	PM_10_	Mouse	5520.0	1	1	1	400.0	400.0	[50]
	PM_10_	Mouse	55200.4	1	5	5	4000.0	4000.0	[44]
	PM_10_	Mouse	55200.4	1	5	5	4000.0	4000.0	[45]
Intratracheal instillation	PM_1_	Mouse	690.0	1	1	1	220.0	220.0	[81]
	PM_1_	Mouse	1254.6	1	1	1	400.0	400.0	[81]
	PM_1_	Mouse	1380.0	1	1	1	440.0	440.0	[81]
	PM_1_	Mouse	1505.5	1	1	1	480.0	480.0	[81]
	PM_1_	Mouse	1568.2	1	7	7	500.0	500.0	[73]
	PM_1_	Mouse	2509.1	1	1	1	800.0	800.0	[81]
	PM_1_	Mouse	3010.9	1	1	1	960.0	960.0	[81]
	PM_1_	Mouse	4390.9	1	2	2	1400.0	1400.0	[66]
	PM_1_	Mouse	12545.6	1	2	2	4000.0	4000.0	[66]
	PM_1_	Mouse	31363.9	1	2	2	10000.0	10000.0	[66]
	PM_1_	Rat	35944.3	1	1	1	5000.0	5000.0	[86]
	PM_2.5_	Rat	278.3	1	1	1	31.6	31.6	[59]
	PM_2.5_	Rat	705.5	1	1	1	80.0	80.0	[68]
	PM_2.5_	Rat	1763.7	1	1	1	200.0	200.0	[59]
	PM_2.5_	Rat	1763.7	1	1	1	200.0	200.0	[59]
	PM_2.5_	Rat	1763.7	1	20	20	200.0	200.0	[71]
	PM_2.5_	Rat	1763.7	1	20	20	200.0	200.0	[79]
	PM_2.5_	Rat	2645.5	1	20	20	300.0	300.0	[71]
	PM_2.5_	Rat	2645.5	1	20	20	300.0	300.0	[79]
	PM_2.5_	Mouse	3295.5	1	4	4	800.0	800.0	[67]
	PM_2.5_	Mouse	3295.5	1	7	7	800.0	800.0	[69]
	PM_2.5_	Rat	3527.3	1	1	1	400.0	400.0	[59]
	PM_2.5_	Rat	3527.3	1	1	1	400.0	400.0	[60]
	PM_2.5_	Rat	3880.1	1	1	1	440.0	440.0	[35]
	PM_2.5_	Rat	5291.0	1	20	20	600.0	600.0	[71]
	PM_2.5_	Rat	5291.0	1	20	20	600.0	600.0	[79]
	PM_2.5_	Mouse	5767.2	1	2	2	1400.0	1400.0	[66]
	PM_2.5_	Rat	8218.7	1	1	1	932.0	932.0	[59]
	PM_2.5_	Rat	8849.7	1	1	1	1003.6	1003.6	[59]
	PM_2.5_	Rat	9982.4	1	1	1	1132.0	1132.0	[59]
	PM_2.5_	Mouse	10298.6	1	1	1	2500.0	2500.0	[65]
	PM_2.5_	Mouse	12358.3	1	3	3	3000.0	3000.0	[76]
	PM_2.5_	Rat	13227.5	1	20	20	1500.0	1500.0	[71]
	PM_2.5_	Rat	13227.5	1	20	20	1500.0	1500.0	[79]
	PM_2.5_	Rat	13227.5	1	3	3	1500.0	1500.0	[84]
	PM_2.5_	Rat	15873.0	1	10	10	1800.0	1800.0	[85]
	PM_2.5_	Mouse	16477.7	1	4	4	4000.0	4000.0	[64]
	PM_2.5_	Mouse	16477.7	1	2	2	4000.0	4000.0	[66]
	PM_2.5_	Mouse	16477.7	1	3	3	4000.0	4000.0	[78]
	PM_2.5_	Rat	17636.7	1	1	1	2000.0	2000.0	[80]
	PM_2.5_	Rat	23809.5	1	20	20	2700.0	2700.0	[71]
	PM_2.5_	Rat	23809.5	1	20	20	2700.0	2700.0	[79]
	PM_2.5_	Rat	26455.0	1	1	1	3000.0	3000.0	[36]
	PM_2.5_	Mouse	27023.5	1	4	4	6560.0	6560.0	[67]
	PM_2.5_	Mouse	32955.5	1	6	6	8000.0	8000.0	[75]
	PM_2.5_	Mouse	32955.5	1	6	6	8000.0	8000.0	[2]
	PM_2.5_	Mouse	32955.5	1	6	6	8000.0	8000.0	[74]
	PM_2.5_	Mouse	41194.4	1	11	11	10000.0	10000.0	[61]
	PM_2.5_	Mouse	41194.4	1	1	1	10000.0	10000.0	[62]
	PM_2.5_	Mouse	41194.4	1	1	1	10000.0	10000.0	[65]
	PM_2.5_	Mouse	41194.4	1	2	2	10000.0	10000.0	[66]
	PM_2.5_	Mouse	41194.4	1	3	3	10000.0	10000.0	[76]
	PM_2.5_	Rat	47619.0	1	10	10	5400.0	5400.0	[85]
	PM_2.5_	Mouse	61791.5	1	5	5	15000.0	15000.0	[70]
	PM_2.5_	Rat	79365.1	1	49	49	9000.0	9000.0	[83]
	PM_2.5_	Mouse	82388.7	1	1	1	20000.0	20000.0	[65]
	PM_2.5_	Rat	88183.4	1	1	1	10000.0	10000.0	[57]
	PM_2.5_	Rat	88183.4	1	1	1	10000.0	10000.0	[77]
	PM_2.5_	Mouse	123583.1	1	3	3	30000.0	30000.0	[76]
	PM_2.5_	Rat	132275.1	1	2	2	15000.0	15000.0	[72]
	PM_2.5_	Rat	142857.1	1	10	10	16200.0	16200.0	[85]
	PM_2.5_	Rat	176366.8	1	1	1	20000.0	20000.0	[77]
	PM_2.5_	Rat	211640.2	1	49	49	24000.0	24000.0	[83]
	PM_2.5_	Rat	220458.6	1	12	12	25000.0	25000.0	[82]
	PM_10_	Rat	5291.0	1	1	1	200.0	200.0	[26]
	PM_10_	Rat	13227.5	1	1	1	500.0	500.0	[56]
	PM_10_	Mouse	19320.2	1	2	2	1400.0	1400.0	[66]
	PM_10_	Rat	21164.0	1	1	1	800.0	800.0	[26]
	PM_10_	Mouse	55200.4	1	1	1	4000.0	4000.0	[58]
	PM_10_	Mouse	55200.4	1	2	2	4000.0	4000.0	[66]
	PM_10_	Rat	66137.6	1	1	1	2500.0	2500.0	[56]
	PM_10_	Rat	79365.1	1	1	1	3000.0	3000.0	[26]
	PM_10_	Mouse	110400.9	1		1	8000.0	8000.0	[63]
	PM_10_	Rat	132275.1	1	1	1	5000.0	5000.0	[56]
	PM_10_	Mouse	138001.1	1	1	1	10000.0	10000.0	[62]
	PM_10_	Mouse	138001.1	1	2	2	10000.0	10000.0	[66]

EAP: environmental air PM, CAP: concentrated air PM.

**Table 4:** Summary of exposure characteristics from different PM exposure protocols in mice and rats.

Exposure Method	PM size	n	Concentration (μg/m^3^)			PM inhalation rate (μg/kg *Bw*/h)		
			Mean	SEM	Range	Mean	SEM	Range
EAP inhalation	PM_2.5_	1	1.8 × 10^1^			4.4 × 10^0^		
	PM_10_	8	8.9 × 10^1^	2.5 × 10^1^	2.2 × 10^1^–2.2 × 10^2^	3.7 × 10^0^	9.0 × 10^1^	8.0 × 10^−1^ - 8.5 × 10^0^
CAP inhalation	PM_1_	3	6.6 × 10^1^	1.2 × 10^1^	5.0 × 10^1^–9.0 × 10^1^	1.2 × 10^1^	2.3 × 10^0^	7.9 × 10^0^–1.6 × 10^1^
	PM_2.5_	41	2.0 × 10^3^	8.9 × 10^2^	6.1 × 10^1^–3.0 × 10^4^	4.0 × 10^2^	2.1 × 10^2^	1.4 × 10^1^–7.3 × 10^3^
	PM_10_	3	3.7 × 10^3^	3.2 × 10^3^	1.0 × 10^2^–1.0 × 10^4^	1.4 × 10^2^	1.2 × 10^2^	3.8 × 10^0^–3.8 × 10^2^
Intranasal instillation	PM_2.5_	18	3.6 × 10^4^	1.5 × 10^4^	7.0 × 10^2^–1.8 × 10^5^	7.8 × 10^3^	3.0 × 10^3^	1.7 × 10^2^–4.0 × 10^4^
	PM_10_	5	2.3 × 10^4^	1.3 × 10^4^	5.5 × 10^1^–5.5 × 10^4^	1.7 × 10^3^	9.5 × 10^2^	4.0 × 10^0^–4.0 × 10^3^
Intratracheal instillation	PM_1_	11	8.7 × 10^3^	3.9 × 10^3^	6.9 × 10^2^–3.6 × 10^4^	2.2 × 10^3^	9.2 × 10^2^	2.2 × 10^2^–1.0 × 10^4^
	PM_2.5_	53	3.9 × 10^4^	7.3 × 10^3^	2.8 × 10^2^–2.2 × 10^5^	6.1 × 10^3^	1.0 × 10^3^	3.2 × 10^1^–3.0 × 10^3^
	PM_10_	12	6.9 × 10^4^	1.4 × 10^4^	5.3 × 10^3^–1.4 × 10^5^	4.1 × 10^3^	1.0 × 10^3^	2.0 × 10^2^–1.0 × 10^4^

These data are a compilation of results in our dataset, as presented in Table 2, Table 3. n: number of experiments, EAP: environmental air PM, CAP: concentrated air PM, SEM: standard error of the mean.

## 2 Experimental design, materials, and methods

### 2.1 Criteria for paper selection

The published works included in our dataset were selected using the following criteria: papers were published in English, they were referenced in indexed journals (with editorial board, peer reviewed and included in Clarivate Analytics *Journal Citation Reports*), and they were published recently (in 2017). The selected papers were based on mice and/or rat models, and the protocol exposed the respiratory tract to PM in order to study the health effects at one or more specific endpoints (respiratory tract, pulmonary and extra-pulmonary). With these criteria, a search query was constructed for PubMed. Searches were performed using the following keywords: (particulate matter) AND (mice or mouse or rats or rat) AND (inhalation or instillation). Apart from the most recent articles published in 2017, we also included seminal papers from previous years.

**Fig. 1:**
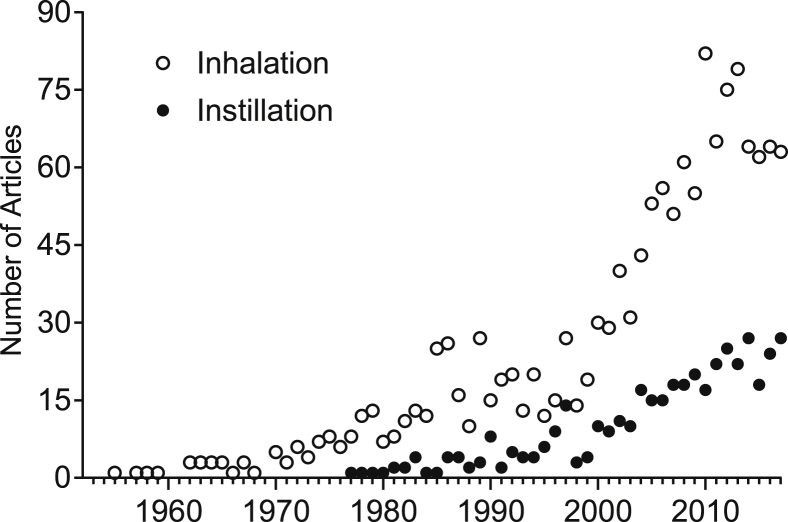
PubMed timelines of two datasets considering the number of articles indexed by “inhalation” and “instillation” as keywords in combination with (particulate matter) AND (rat or rats or mouse or mice). The timeline begins at 1955 (first “inhalation” article found) and ends at 2017.

From the PubMed timeline of the selected papers (Fig. 1), an increase can be observed in the number of publications indexed by the keywords “inhalation” and “instillation” with “inhalation” cited in more articles than “instillation”. Such behaviour is similar over other years.

From the resulting group of articles, only those papers containing experimental impacts of environmental PM effects, as described in their material and methods sections, were included. Experiments that used total suspended particles or settleable particulate matter (six articles) and experiments carried out by oropharyngeal aspiration (four articles) were excluded. After applying the filtering criteria to the PubMed query results, 75 articles were selected, containing 155 different experiments.

Normalization of different experimental results to allow comparisons among doses and results.

One difficulty in comparing studies that use different exposure protocols is the way as PM amounts are reported. Studies using instillation protocols report the mass of administered PM (dose) at each exposure event and studies using inhalation, report PM concentration and exposure time. To compare data and results for methods having such intrinsic differences, we used an equation based on PM dosimetry models [3], [4], [5]. This equation normalizes the administered PM dose (*D*), taking into account PM inhaled fraction (inhalability) as a function of the aerodynamic diameter (*d*_a_) [3], [4], [5]. Thus, the following formula was defined:

(1) D=C⋅Q⋅t⋅I

The parameters used in this formula are described below:

*D* = dose according to *d*_*a*_ (μg)

*C* = PM concentration (μg/m^3^)

*Q* = air inhalation flow (m^3^/min)

*t* = exposure time (min)

*I* = inhalability according to *d*_*a*_ (%)

The exposure dose varies according to the respiratory physiologic parameters of each species (Table 1), which are experimentally obtained or calculated by allometric models [4], [6], [7]. The air inhalation flow (*Q*) is calculated from the ratio between the tidal volume (*V*_*T*_) and the inhalation time (*t*_*i*_) and indicates the inhaled air volume per unit of time [8]. Assuming a ratio of 0.4/0.6 for inhalation versus exhalation times in mice and rats [9], [10], it is possible to estimate the *t*_*i*_ according to the respiration frequency (*f*) in mice and rats. We defined the PM inhaled dose as the PM mass that reaches the respiratory tract, i.e., it is the dose that can be inhaled, even if part of PM were deposited in the upper respiratory tract (URT). This definition is consistent with the concept of delivered dose, the amount of PM inhaled by the animal [11].

This equation is not only useful for calculating the administered PM doses, but can also be used to normalize other quantities and to calculate the equivalent PM concentration (C), according to the instillation protocols (*C* = *D*/(*Q·t·I*)). For example, the PM inhalation rate (*IR*, μg/h) can be determined if we disregard exposure time (*t*). Moreover, both *D* and *IR* can be expressed in terms of the experimental animal body mass, and in these cases, two other indices are determined: specific dose (*D*_*BW*_) and specific inhalation rate (*IR*_*BW*_).

Since we have different exposure protocols, some considerations about the application of equation (1) are necessary:

(i) Equation (1) was used to establish the equivalence between protocols performed by instillation and inhalation. The protocols by instillation are performed after anaesthetics administration, which can change the physiological breathing parameters of the animals. However, equation (1), does not consider the effect of anaesthesia. Thus, the variations of the breathing parameters that could be caused by anaesthesia are not included in applying equation (1), designed to calculate the delivered dose by inhalation.

(ii) Physiological breathing parameters described in Table 1 may present variations according to the different references. These parameters can vary between species, strains and even between individuals. However, within the limits of application of equation (1), we define the data acquisition referenced in recent publications developed by specialist researchers. At present, order of magnitude parameter estimation is the most relevant way to study different methods because the variations of PM exposure between methods are quite large and exceed orders of magnitude. Thus, variations in the physiological breathing parameters within the same order of magnitude can be assimilated without changing the conclusions of this study.

(iii) In instillation protocols, the PM is administered in a liquid media, while in the inhalation methods, the PM is dispersed in air. The distribution and deposition of PM in the respiratory tract is different when administered in liquid or in the air. It is known that intranasal instillation (INI), even in a liquid media, must pass the URT before reaching the LRT. However, considering the definition of inhaled dose previously mentioned, in this study, even if the deposition occurs in URT, we consider that the respiratory tract had contact with PM. Some studies included in our dataset, examined extra-pulmonary and systemic PM effects, including allergic responses in URT [12], [13]. In addition, instillation protocols introduce into airways types and quantities of particles that would not naturally reach there by inhalation. This may be one of the most relevant differences between exposure methods. Thus, inhalability was used as an important variable to calculate the equivalence between instillation and inhalation methods. As such, inhalability considers the difficulty of reaching the airways imposed by aerodynamic restrictions related to particle size [3], [14].

(iv) Finally, in some cases, few assumptions should be pre-determined to allow the calculation of *D* and *C*. In studies based on instillation, the amount of time spent to perform this procedure was 1 h. We assumed this time duration to calculate the dosage used. In fact, this duration is smaller than 1 h, probably minutes, but this would raise *D* and *C* to very excessive doses when compared with environmental concentrations measured around the world. In addition, we used a 1 h dose duration because most air quality monitoring stations use 1 h as the shortest interval for recording measurements. Thus, based on this average time, we could compare the concentrations used in the experiments and the measured environmental concentrations. In fact, the instillation protocols try to mimic in minutes (or less time) the exposure that would occur in hours, days, even months in environmental conditions.
